# Long noncoding RNAs, emerging players in muscle differentiation and disease

**DOI:** 10.1186/2044-5040-4-8

**Published:** 2014-03-31

**Authors:** Maria Victoria Neguembor, Mathivanan Jothi, Davide Gabellini

**Affiliations:** 1 Dulbecco Telethon Institute at San Raffaele Scientific Institute, Division of Regenerative Medicine, Stem cells, and Gene therapy, DIBIT2, 5A3, Via Olgettina 58, 20132 Milano, Italy

**Keywords:** Chromatin, DMD, FSHD, Muscular dystrophy, ncRNA, Repeat

## Abstract

The vast majority of the mammalian genome is transcribed giving rise to many different types of noncoding RNAs. Among them, long noncoding RNAs are the most numerous and functionally versatile class. Indeed, the lncRNA repertoire might be as rich as the proteome. LncRNAs have emerged as key regulators of gene expression at multiple levels. They play important roles in the regulation of development, differentiation and maintenance of cell identity and they also contribute to disease. In this review, we present recent advances in the biology of lncRNAs in muscle development and differentiation. We will also discuss the contribution of lncRNAs to muscle disease with a particular focus on Duchenne and facioscapulohumeral muscular dystrophies.

## Review

### Long non protein-coding RNAs (lncRNAs)

In mammals, the vast majority of the transcriptional output is noncoding [1]. While 75% of the genome is transcribed, only 2% encodes for proteins [2]. Non protein-coding RNAs (ncRNAs) are operationally divided in two classes according to their size. Small ncRNAs are below 200 bp and include transfer RNA (tRNA), ribosomal RNA (rRNA), small nuclear RNAs (snRNA), small nucleolar RNAs (snoRNA), microRNAs, siRNAs and Piwi-interacting RNAs (piRNA) [3-7]. Long ncRNAs (lncRNAs) include all ncRNA transcripts greater than 200 bp with little or no coding potential. Although discovered relatively recently, lncRNAs are considered the most numerous and functionally diverse class of RNAs [8]. Up to 15,000 lncRNAs have been identified so far [9] and, as the number constantly increases, the lncRNA assortment might turn out to be as rich as the proteome.

LncRNAs loci are often in close association with protein-coding genes as they are encoded from exonic or intronic sequences in both sense and antisense orientation or even from gene regulatory regions [10]. LncRNAs can also arise from intergenic regions including repetitive sequences [11]. Most lncRNAs are transcribed by RNA polymerase II and may share mRNA-like features such as 5’cap, polyA tail and splicing sites [12,13]. Alternatively, non-polyadenylated lncRNAs are likely generated by RNA polymerase III [14,15].

In terms of transcriptional profile, lncRNAs are generally expressed at lower levels than protein-coding transcripts and, compared to the latter, their pattern of expression is more developmental stage- and cell type-specific [2,16]. The intrinsic nature and complex secondary structures of lncRNAs enable them to specifically interact with DNA, RNA and proteins. Since lncRNAs are localized both in the nucleus and the cytosol, they can act at virtually every level of gene expression [17,18].

### LncRNA, a molecular ‘passepartout’

LncRNAs can be divided into multiple functional categories based on the site of action and the level of gene expression at which they act. However, as our knowledge of lncRNAs increases, new functional groups emerge and the distinction between classes is not always adequate. Here, we present a very brief classification to provide a framework for the examples of lncRNAs acting in muscle differentiation and disease later described.

**Figure 1:**
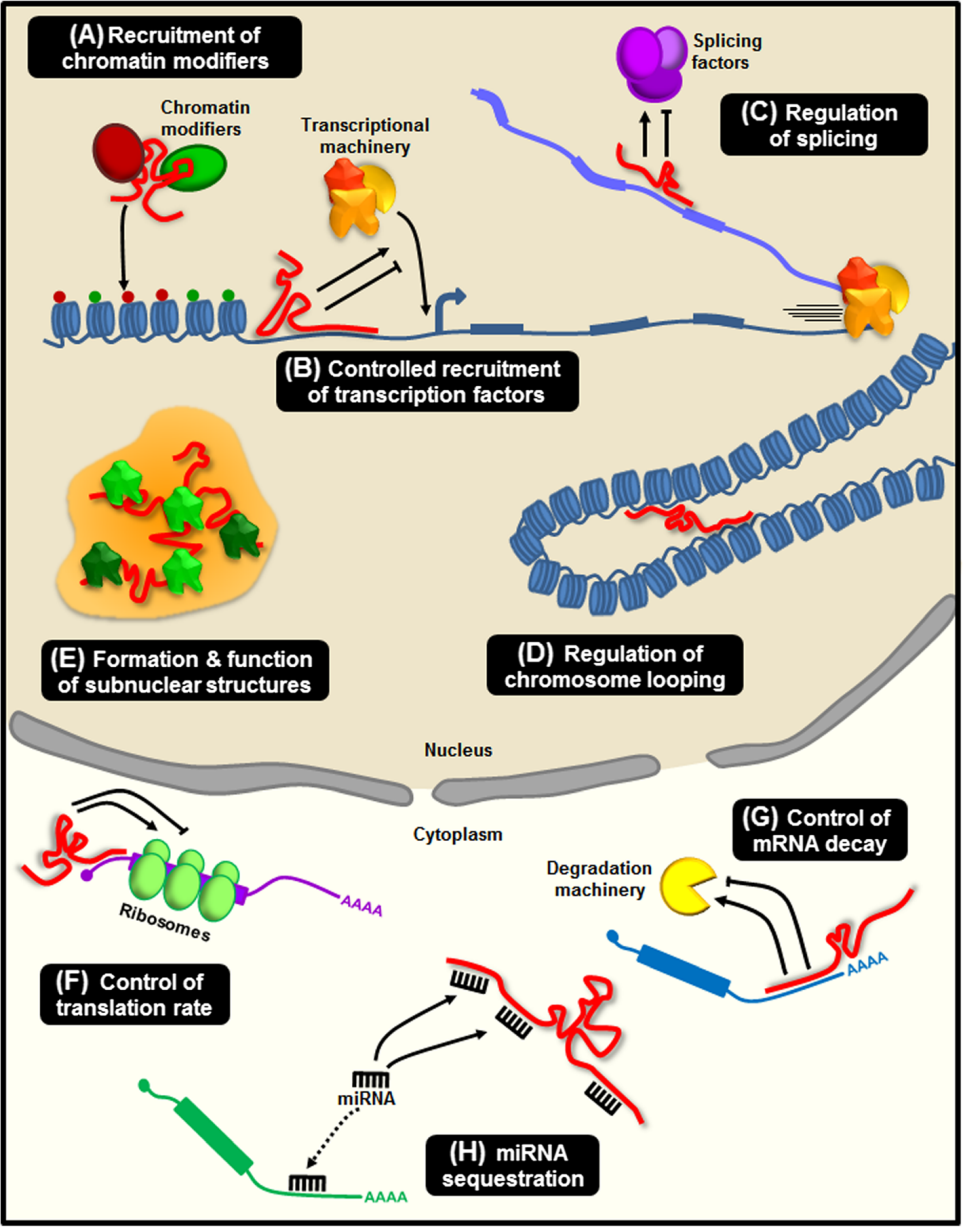
**Mechanisms for long noncoding RNA (lncRNA) function. (A)** LncRNAs (in red) are able to recruit chromatin modifiers mediating the deposition of activatory (green dots) or repressive (red dots) histone marks. **(B)** LncRNAs control the recruitment of transcription factors and core components of the transcriptional machinery. **(C)** LncRNAs can directly bind mRNAs and modulate splicing events. **(D-E)** LncRNAs participate in the higher order organization of the nucleus by mediating chromatin looping **(D)** and as structural components for the formation and function of nuclear bodies **(E). (F)** LncRNAs control translation rates favoring or inhibiting polysome loading to mRNAs. **(G)** LncRNAs modulate mRNA decay protecting mRNA from degradation or, alternatively, mediating the recruitment of degradation machinery. **(H)** LncRNAs can act as miRNA sponges, thus favoring the expression of the mRNAs targeted by the sequestered miRNA.

Nuclear lncRNAs can be subdivided into *cis*-acting RNAs that work in proximity to their site of transcription, and *trans*-acting RNAs that operate at distant loci. Both *cis*- and *trans*-acting lncRNAs can activate or repress transcription through the recruitment of chromatin remodelers and modifiers, thus shaping the chromatin status of a particular locus or even of an entire chromosome (Figure 1A) [19-24]. Besides, lncRNAs are able to recruit or prevent the binding of the transcriptional machinery and transcription factors directly impacting the transcriptional output of a region (Figure 1B) [25-28]. Among these, enhancer RNAs (eRNAs), are encoded by extragenic enhancer regions and promote transcription of surrounding genes [29-32]. LncRNAs also participate in co- and post-transcriptional regulation in the nucleus. For example, lncRNAs can interact with the splicing machinery or directly with nascent mRNAs to guide particular splicing events (Figure 1C) [33-35]. In addition to the *cis* versus *trans* distinction, lncRNAs can shape the subnuclear architecture in different ways. Certain lncRNAs regulate chromosome looping, favoring or disrupting chromosomal interactions (Figure 1D) [36,37]. Others act as structural scaffolds for the formation and regulation of nuclear compartments such as speckles [33], paraspeckles [38] and Polycomb bodies [39] (Figure 1E).

In the cytoplasm, lncRNAs influence translational output in different ways. Firstly, they can modulate the translational rate by regulating polysome loading to an mRNA molecule (Figure 1F) or through the control of internal ribosomal entry sites (IRES) [40-42]. Secondly, they can regulate gene expression by reducing or stimulating mRNA decay (Figure 1G) [43,44]. A particular class of cytoplasmic lncRNAs, the competing endogenous RNAs (ceRNA), regulates both the translation and the degradation rates of mRNAs by acting as molecular sponges for miRNAs, thus modulating the repressive activity of miRNA on their mRNA targets (Figure 1H) [45-49].

Altogether, lncRNAs exhibit remarkable functional flexibility and tightly regulated expression that confer on them an enormous potential as fine tuners of cell function and identity. Due to their versatility, they are able to control different aspects of cellular development, from stem cell maintenance to commitment and differentiation and we anticipate their biological role in a great variety of cell types to be uncovered in the near future [50]. In this review, we will focus on the fascinating discoveries regarding the role of lncRNAs in muscle differentiation and disease, with a particular focus on Duchenne muscular dystrophy (DMD) and facioscapulohumeral muscular dystrophy (FSHD).

### LncRNAs in myogenic differentiation

Myogenesis is the process where progenitor cells give rise to myoblasts that fuse onto multi-nucleated myofibers endowed with contractile ability. This complex and tightly regulated process starts from extra/intra cellular signals impinging on the myogenic transcription program. An enormous literature provides evidence that the myogenic gene expression program is orchestrated by a transcriptional hierarchy, including the Myogenic Regulatory Factors (MyoD, Myf5, Myogenin, and MRF4) and the Myocyte Enhancer Factor-2 (MEF2A-D) families of transcription factors (reviewed in [51] and [52]). In a stage-specific manner, these factors act in coordination with other transcriptional regulators, including epigenetic factors, to execute the muscle differentiation program [53]. Still, this scenario is not complete as new players are gradually emerging. Indeed, there is increasing evidence that ncRNAs are also part of the muscle regulatory network. So far, miRNAs are the most extensively studied and characterized [54]. However, in the last years lncRNAs are emerging as critical regulators of muscle differentiation (Table 1).

**Table 1:** Long noncoding RNAs (lncRNAs) involved in muscle differentiation

**LncRNA**	**Site of action**	**Function**	**Effector molecule**	**Regulation during muscle differentiation**	**References**
eRNAs (^*CE* and *DRR*^*RNAs*)	Nucleus	Transcriptional activation	MyoD	Up	[30]
*Gtl2/Meg3*	Nucleus	Epigenetic repression	PRC2	Up	[55,56]
*H19*	Nucleus and cytoplasm	Epigenetic repression, miRNAs sponge	PRC2, let-7 miRNAs	Up	[57]
*Linc-MD1*	Cytoplasm	miRNAs sponge	HuR	Up	[46,58]
*Malat1*	Nucleus	Epigenetic repression, pre-mRNA splicing	Cbx4 and SR family of splicing factors	Up	[59]
*Neat1*	Nucleus	Structural integrity of nuclear paraspeckles	Various RNA-binding proteins	Up	[60]
*Nctc1*	Nucleus?	Unknown	Unknown	Up	[61]
*SRA*	Nucleus	Scaffold factor	MyoD	Up	[62,63]
SINE containing lncRNAs	Cytoplasm	mRNA decay	STAU1 and STAU2	Up	[64]
*Yams*	Nucleus	Transcriptional activation	Unknown	Up/down	[65]

eRNAs: enhancer RNAs; ^*CE* and *DRR*^*RNAs*: Core Enhancer and Distal Regulatory Region RNAs; *Gtl2/Meg3*: Gene trap locus 2/Maternally expressed gene 3; *Malat1*: Metastasis associated lung adenocarcinoma transcript 1; *Neat1*: Nuclear enriched abundant transcript 1; *Nctc1*: Noncoding transcript 1; *SRA*: Steroid receptor RNA Activator; SINE containing lncRNAs: Short Interspersed Elements containing lncRNAs; *Yams*: YY1-associated muscle lincRNAs.

### LncRNAs mediating the activity of chromatin modifiers and transcription factors

**Figure 2:**
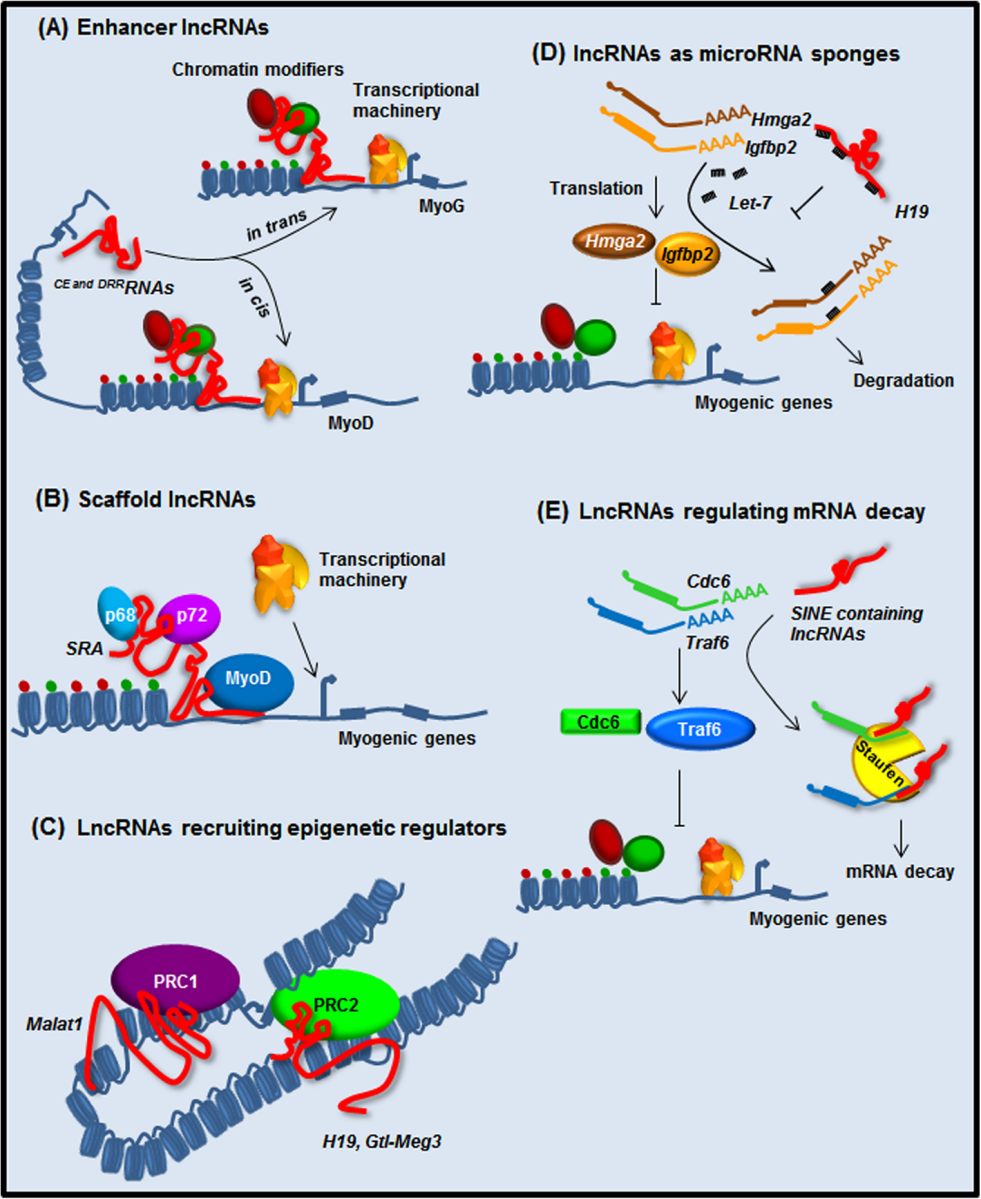
**Distinct roles of long noncoding RNAs (lncRNAs) in muscle differentiation. (A)** Enhancer RNAs (eRNAs) ^*CE* and *DRR*^*RNAs* can induce expression of myogenic regulators *MyoD* and *MyoG* acting *in cis* or *in trans*, respectively. **(B)** LncRNA *SRA* acts as a scaffold molecule for MyoD, p68 and p72 at the promoter region of myogenic genes to activate their expression. **(C)** LncRNAs *Malat1*, *H19* and *Gtl-Meg3* interact with PRC1/2 complex to modulate their target genes. **(D)** As a molecular sponge *H19* inhibits *let-7* mediated mRNA degradation of myogenic negative regulators *Hmga2 and Igfbp2.***(E)** Short interspersed element (SINE) containing lncRNAs can bind to UTR region of *Cdc6* and *Traf6* mRNAs and promote their decay at different stages of muscle differentiation.

An increasing body of work indicates that many nuclear lncRNAs regulate the activity of enhancers at various levels. Enhancers are distal regulatory elements that play an essential role for the proper temporal and tissue-specific expression of protein-coding genes. Typically, active enhancers display increased chromatin accessibility and are enriched for monomethyl histone H3 lysine 4 (H3K4me1) and acetylated H3K27 (H3K27ac). Intriguingly, RNA polymerase II (RNAPII) is also enriched at active enhancers where it drives localized transcription of lncRNAs called enhancer-derived RNAs (eRNAs) [31,66]. Many eRNAs regulate enhancer/promoter communication by directly recruiting chromatin modifiers and remodelers and the transcriptional machinery, thus favoring the activation of gene expression *in cis* or *in trans* (Figure 1B) [29,32,36,67-70]. Recently, a key role for eRNAs in the regulation of muscle differentiation was uncovered [30]. In myotubes, ChIP-seq analyses revealed that the myogenic regulatory factors MyoD and MyoG display a very similar genome-wide binding profile and are mostly associated with extragenic regions, many of which exhibit eRNA features [30]. Several of these eRNAs are preferentially localized to the nucleus and are mainly regulated by MyoD, as MyoG silencing has a marginal effect on their expression [30]. In turn, two eRNAs generated by upstream regulatory regions of *MyoD* (*CE* and *DRR*) regulate the expression of *MyoD* and *MyoG* (Figure 2A and Table 1) [71,72]. Both eRNAs are involved in the activation of gene expression, but they differ in their mode of action. While the ^*CE*^*RNA* functions *in cis* to activate expression of *MyoD*, ^*DRR*^*RNA* works *in trans* to promote *MyoG* transcription and muscle differentiation (Figure 2A). At their site of action, both eRNAs mediate increased chromatin accessibility and recruitment of RNAPII [30]. Collectively, these findings suggest that eRNAs regulate myogenesis by directing chromatin-remodeling events, controlling the hierarchy within the myogenic gene regulatory network.

The transcription factor Yin Yang 1 (YY1) is another important regulator of myogenesis at multiple levels [73-77]. By comparing YY1 ChIP-seq results in proliferating and differentiated C2C12 muscle cells, a number of lncRNAs regulated by YY1 (*YY1-associated muscle lincRNAs* or *Yams*) have been recently identified [65]. *Yams* display distinct expression patterns during muscle differentiation and affect myogenesis differently (Table1). For example, while *Yam-2* and *-3* promote C2C12 muscle differentiation, *Yam-1* and *-4* inhibit it [65]. The mechanism of action of *Yam-1* was investigated in more detail. *Yam-1* is a single exon transcript, regulated during *in vitro* and *in vivo* myogenesis and equally distributed in the nucleus and cytoplasm. Throughout development, in muscle regeneration and in tissue culture, *Yam-1* is downregulated during terminal muscle differentiation [65]. *Yam-1* knockdown promotes muscle differentiation in tissue culture and accelerates regeneration following muscle injury *in vivo*[65]. Importantly, *Yam-1* knockdown rescues the myogenic defects caused by YY1 overexpression indicating that *Yam-1* is an important mediator of YY1 activity in muscle [65]. Silencing of *Yam-1* reduces the expression of several nearby genes, suggesting that it could be a positive *cis*-regulator of surrounding genes similar to eRNAs (Figure 1B) [65]. One of the nearby genes co-regulated with *Yam-1* encodes for *miR-715*, a miRNA targeting *Wnt7b*. Since Wnt signaling is a critical modulator of skeletal muscle formation [78], it is tempting to speculate that *Yam-1* could function, at least in part, by activating *miR-715 in cis* leading to *Wnt7b* downregulation.

Besides regulating chromatin accessibility like eRNAs, nuclear lncRNAs can also control gene expression by directly affecting the activity of sequence-specific transcription factors. This is the case for *steroid receptor RNA activator* (*SRA*) [79], the first example of lncRNA regulating myogenesis [62]. *SRA* likely functions as a scaffold, bringing together multiple factors that modulate gene expression [80] including the master regulator of muscle differentiation MyoD (Figure 2B and Table1) [62]. In muscle, a complex composed by *SRA*, MyoD and the RNA helicases p68 and p72 has been identified (Figure 2B) [62]. Knockdown and overexpression studies indicate that p68/p72 and *SRA* are coactivators required for the transcription of a subset of MyoD target genes and for muscle differentiation [62]. The *SRA* gene produces multiple transcripts through alternative splicing. While retention of intron 1 gives rise to the non protein-coding *SRA* transcript, splicing of the intron creates an open reading frame that generates the SRA protein (SRAP) [81]. The ratio between the coding and noncoding *SRA* transcripts varies during muscle differentiation with *SRAP* mRNA being more abundant in myoblasts and *SRA* lncRNA being the predominant isoform in myotubes [63]. SRAP works at least in part by regulating *SRA* lncRNA coactivator function. Indeed, SRAP is an RNA-binding protein that specifically binds *SRA* lncRNA, thus preventing *SRA*-mediated regulation of MyoD transcriptional activity [63]. Hence, the correct balance between coding and noncoding *SRA* molecules is important for normal muscle differentiation. Intriguingly, aberrant *SRA* splicing is present in myotonic dystrophy patients. Whether impaired splicing of *SRA* contributes to the pathogenesis of muscular dystrophy remains to be elucidated [63].

### Subnuclear structure-specific lncRNAs

A number of lncRNAs are enriched in and contribute to organize specific subnuclear domains (Figure 1E) [82-88]. The lncRNA *Malat1* is enriched in nuclear speckles, abundantly expressed in cancer cells and a strong predictor of tumor metastasis [89]. *Malat1* has been shown to regulate gene transcription and pre-mRNA splicing by respectively interacting with the epigenetic repressor Polycomb protein Cbx4 [39] and with the SR family of splicing factors (Figure 2C and Table 1) [33]. *Malat1* is upregulated during early differentiation of C2C12 mouse myoblasts and primary human skeletal muscle cells, while its knockdown leads to suppression of myoblast proliferation by arresting cells in the G0/G1 phase [59], suggesting a role for *Malat1* in the transition from the proliferative phase to the permanent cell cycle exit, as well as in the commitment to differentiation. Intriguingly, *Malat1* has been recently identified as a novel downstream target of myostatin [59], an important regulator of myoblast proliferation, differentiation and skeletal muscle mass [90]. Future work will indicate how relevant *Malat1* is for myostatin activity.

### Imprinted lncRNAs

The *Dlk1-Dio3* region is a very complex, imprinted locus involved in tissue growth regulation and human cancers [91]. Aberrant repression of the *Dlk1-Dio3* imprinted cluster is present in most induced pluripotent stem cell (iPSC) lines and is responsible for the failure of iPSCs to form viable mice [92]. Intriguingly, postnatal aberrant expression of this locus is responsible for muscle hypertrophy in mouse and sheep [93,94]. The locus contains protein-coding RNAs, lncRNAs, miRNAs and snoRNAs expressed from either the paternal or the maternal allele. Several of the lncRNAs transcribed from the *Dlk1-Dio3* region are enriched in the nucleus and have been reported to bind to Polycomb Repressive Complex 1 (PRC1), PRC2 and other epigenetic repressors [95-97]. Expression of the various *Dlk1-Dio3* transcripts is reciprocally regulated. For example, the *Glt2/Meg3* lncRNA binds to PRC2 recruiting it to the *Dlk1-Dio3* locus to repress the protein-coding gene *Dlk1* and the lncRNA *Gtl2-as* (Figure 2C and Table 1). Consistently, *Glt2/Meg3* knockdown leads to a decreased PRC2 recruitment at the Dlk1 promoter with subsequent increased expression of *Dlk1* and *Gtl2-as*[95]. During development, *Gtl2/Meg3* is abundantly expressed in the paraxial mesoderm suggesting a role in myogenesis [55]. Indeed, *Gtl2/Meg3* knockout mice develop skeletal muscle developmental defects along with perinatal death [56], promoting *Gtl2/Meg3* as an example of a nuclear, *cis*-acting lncRNA regulating muscle development.

*H19* was the first lncRNA described in mammalian cells [98]. It is transcribed from the maternal allele of the *H19/Igf2* locus producing a lncRNA predominantly enriched in the cytoplasm [98]. *H19* is highly expressed in developing embryo and adult muscle in human and mouse [99,100], and is upregulated during myoblast differentiation and muscle regeneration [57,101]. *H19* works at multiple levels. Through binding to the PRC2 epigenetic repressor complex [95], *H19* can mediate the transcriptional repression of *Igf2* (Figure 2C) [102,103]*.* Moreover, *H19* can bind *Igf2* mRNA binding-protein (IMP) family members to regulate *Igf2* post-transcriptionally [104]. Also, *H19* contains several binding sites for the *let-7* family of microRNAs suggesting that *H19* may act as a miRNA sponge for *let-7* (Figure 2D and Table 1) [57]. Among *let-7* targets, *Hmga2* and *Igfbp2* have an important role in myoblasts proliferation and myogenesis but must be downregulated to allow the formation of multinucleated myofibers (Figure 2D) [105]. Finally, *H19* exon 1 encodes the conserved microRNAs *miR-675-3p* and *miR-675-5p*[106]. Recent results indicate that, by targeting *Smad1*, *Smad5*, and *Cdc6*, these miRNAs play an important role in the skeletal muscle differentiation and regeneration activities associated to *H19*[101].

### LncRNAs containing repetitive sequences controlling mRNA decay

Short interspersed elements (SINEs) are among the most abundant repetitive sequences in mammalian genomes [107]. While initially thought of as ‘junk’ DNA, SINEs can be transcribed as individual elements by RNA polymerase III or as part of longer transcripts synthesized by RNA polymerase II and they can regulate gene expression by diverse mechanisms [41,108]. It has recently been discovered that a SINE within the 3′ UTR of a protein-coding RNA can form intermolecular base pairing with a partially complementary SINE within one or more lncRNAs [43,64]. Extensive yet imperfect stretches of double-stranded RNA (dsRNA) can be bound at multiple sites by dsRNA-binding proteins, including Staufen 1 and 2 (STAU1 and STAU2). Staufen recruitment activates Staufen-mediated mRNA decay (SMD), an important mRNA degradation process in mammalian cells. Using this mechanism, lncRNAs containing SINEs regulate the stability of several mRNAs encoding for proteins with a role in muscle differentiation, including *Cdc6* and *Traf6* (Figure 2E and Table 1) [64]*.* Both STAU1 and STAU2 interact directly with the ATP-dependent RNA helicase UPF1, a key SMD factor, enhancing its helicase activity to promote effective SMD. Because both SMD and the mechanistically related nonsense-mediated mRNA decay (NMD) employ UPF1, SMD and NMD are competitive pathways. This competition plays an important role in the control of muscle differentiation. Indeed, during myogenesis, the efficiency of NMD decreases while the efficiency of SMD increases. Interestingly, *Myogenin* and *PAX3* are differentially targeted by these two pathways of degradation and this different susceptibility contributes to their relative abundance during differentiation. *PAX3* mRNA is an SMD target and its increased decay promotes myogenesis, whereas decreased degradation of the NMD target *Myogenin* is required for myogenesis [109]. Importantly, since close to one third of all lncRNAs contains at least one SINE [64,110], lncRNAs containing SINE sequences could be at the heart of many physiologically important processes in addition to myogenesis.

### LncRNAs in muscle diseases

Growing evidence shows that the vast majority of disease-associated genetic variations occur in the noncoding portion of the genome. In fact, whereas only 7% of disease-associated SNPs localize in protein-coding exons, the remaining 93% arise in noncoding areas of the genome, of which 43% fall in intergenic regions [111]. Considering the extensive transcription of these areas, it is reasonable to predict that a significant and yet unknown number of lncRNAs are involved in a variety of human diseases. LncRNAs can either have a primary role in the pathogenesis of a disease or rather act as modulators of disease penetrance, explaining, at least in part, the inter-personal variability observed in virtually every disorder. So far, the contribution of lncRNAs to disease has mostly been investigated in cancer and neurological disorders [112-114] but the first examples of lncRNA involved in myopathies are now unveiled. Here, we focus on the recent discoveries regarding the role of lncRNAs in Duchenne muscular dystrophy and facioscapulohumeral muscular dystrophy.

### Duchenne muscular dystrophy

Duchenne muscular dystrophy (DMD) is the most common and severe myopathy affecting 1:3,500 males. It is inherited in an X-linked recessive manner but, in very rare cases, heterozygous females can be mildly affected [115]. DMD is characterized by severe muscle wasting from early childhood that usually arises in leg and pelvic muscles and later extends to the trunk of the body, compromising the heart and respiratory muscles. DMD is caused by a variety of out-of-frame mutations in the *dystrophin* (*DMD*) gene encoded on the X chromosome (Xp21.2) resulting in the lack of a functional dystrophin protein in skeletal muscle. With 79 exons and 2.4 Mb in size, *DMD* is the largest gene of our genome and up to 2,900 types of mutations have been reported in DMD patients so far [116,117]. Despite the mutations triggering the disease being well characterized, the regulation of *dystrophin* is in part unknown and appears much more complex than previously thought. Moreover, the inter-individual variability in the severity and the disease progression is only partially explained by the types of mutations and the occurrence of female patients remains enigmatic. Therefore, the regulation of *DMD* is an area of intense research that in the last few years has led to the characterization of miRNA and lncRNA involvement [118].

**Figure 3:**
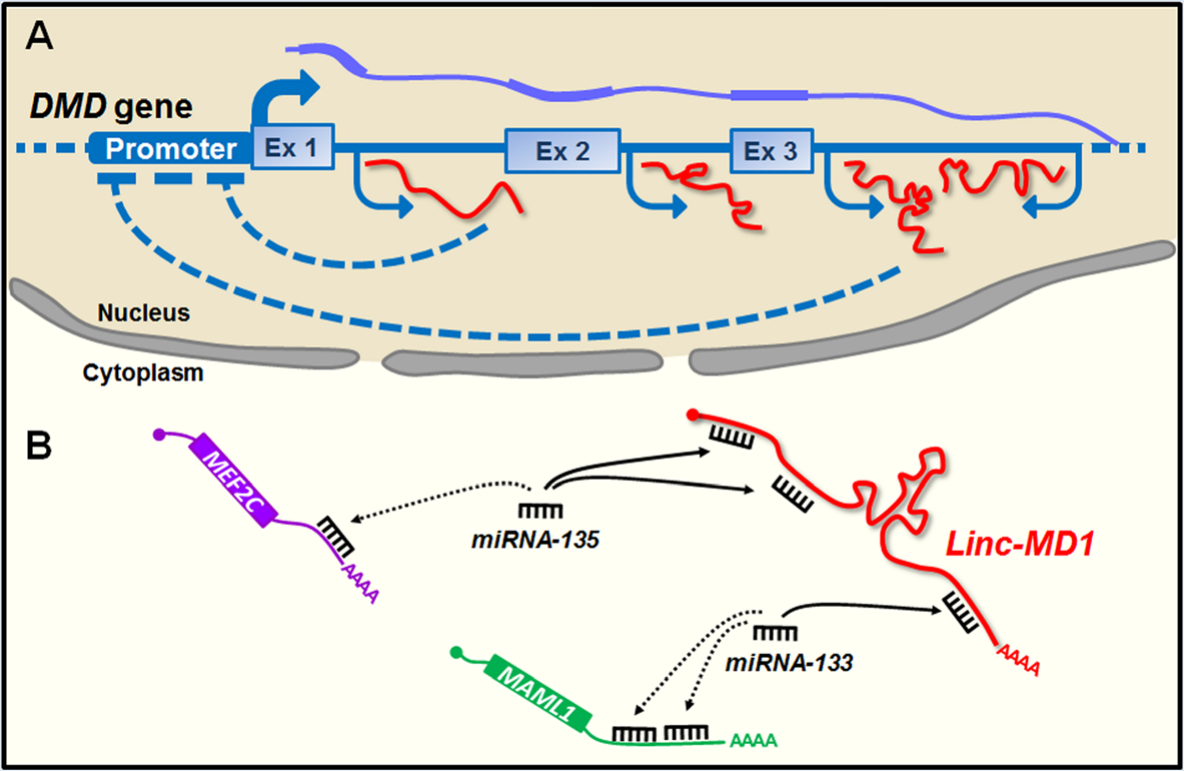
**Proposed roles for long noncoding RNAs (lncRNAs) in Duchenne muscular dystrophy. (A)** In the nucleus, sense and antisense transcription from intronic sequences of the *dystrophin* (*DMD*) gene gives rise to lncRNAs that play a repressive effect at specific *DMD* promoters. **(B)** In the cytoplasm, the muscle specific lncRNA *linc-MD1* acts as a competitive endogenous RNA (ceRNA) by sequestering miRNAs away from their target mRNAs. *Linc-MD1* contributes to muscle differentiation by sponging miRNA-135 and -133, and thus promoting the expression of *MEF2C* and *MAML1. Linc-MD1* is strongly reduced in muscle cells from DMD patients.

Using custom-made tiling arrays, 14 lncRNAs transcribed from intronic sequences of the *DMD* gene both in sense and antisense orientations were recently identified [119]. These lncRNAs are expressed concomitantly with *dystrophin* in at least one of the tissues that normally express it: skeletal muscle, heart and brain [119]. Ectopic expression and promoter binding assays suggest that the lncRNAs can function *in trans* by downregulating the expression of specific *dystrophin* isoforms targeting their promoters (Figure 3A). Interestingly, an inverse correlation was found between the levels of *dystrophin* and a subset of lncRNAs in female carriers, both symptomatic and asymptomatic [119]. It remains to be investigated if these lncRNAs act in an allele-specific manner or whether they may also modulate the expression of the wild type *dystrophin* allele in female carriers. Additionally, it would be interesting to define how different *DMD* mutations may impact the expression of the lncRNAs.

The mutations occurring in DMD patients could also deregulate the expression of lncRNAs located outside the *dystrophin* gene or could even give rise to new lncRNAs as a result of translocation events. This could modulate the severity of the muscle degeneration or contribute to the development of additional symptoms such as the neurological complications observed in around one third of the patients [120,121]. A single case study recently published explores this hypothesis and describes how an intrachromosomal inversion (inv(X)p21.2;q28) disrupts the novel lncRNA *KUCG1* in a DMD patient with moderate mental retardation [122]. *KUCG1* is a 648-bp nuclear lncRNA expressed in a tissue specific manner [122]. Since it is normally expressed in the brain, its deregulation could contribute to the neurological impairment of the patient [122] as already reported for other pathologies [114]. Although a functional characterization of this transcript has not been performed, this study underscores the pathological potential of mutations in noncoding loci that often follow genomic rearrangements.

Another lncRNA associated with DMD is *long intergenic noncoding RNA-muscle differentiation 1* (*linc-MD1*) [46]. *Linc-MD1* is a muscle-specific lncRNA required to activate late stages of the myogenic program. *Linc-MD1* is a cytoplasmic ceRNA that acts as a molecular sponge for *miR-133* and *miRNA-135* (Figure 3B and Table 1) [46]. Through this mechanism, *linc-MD1* promotes the expression of *myocyte-specific enhancer factor 2C (MEF2C)* and *mastermind-like protein 1 (MAML1)*, two transcription factors with an important role in muscle differentiation (Figure 3B) [46]. Interestingly, the levels of *linc-MD1* are strongly reduced in primary myoblasts of DMD patients and its ectopic expression rescues the myogenic differentiation potential of these cells, restoring the correct expression pattern of *MAML1, MEF2C, MYOG* and *MHC*[46]. *Linc-MD1* can have a double life as lncRNA or as miRNA, since its primary transcript harbors the *pri-miR-133b* sequence. The balance between *linc-MD1* and *miR-133* biogenesis is regulated by HuR, an RNA-binding protein with a crucial role in myogenesis (Table 1) [123]. Moreover, HuR facilitates the *linc-MD1*-miRNA interaction, enhancing its sponge activity, thus affecting this ceRNA circuitry potentially relevant for DMD [58].

### Facioscapulohumeral muscular dystrophy

**Figure 4:**
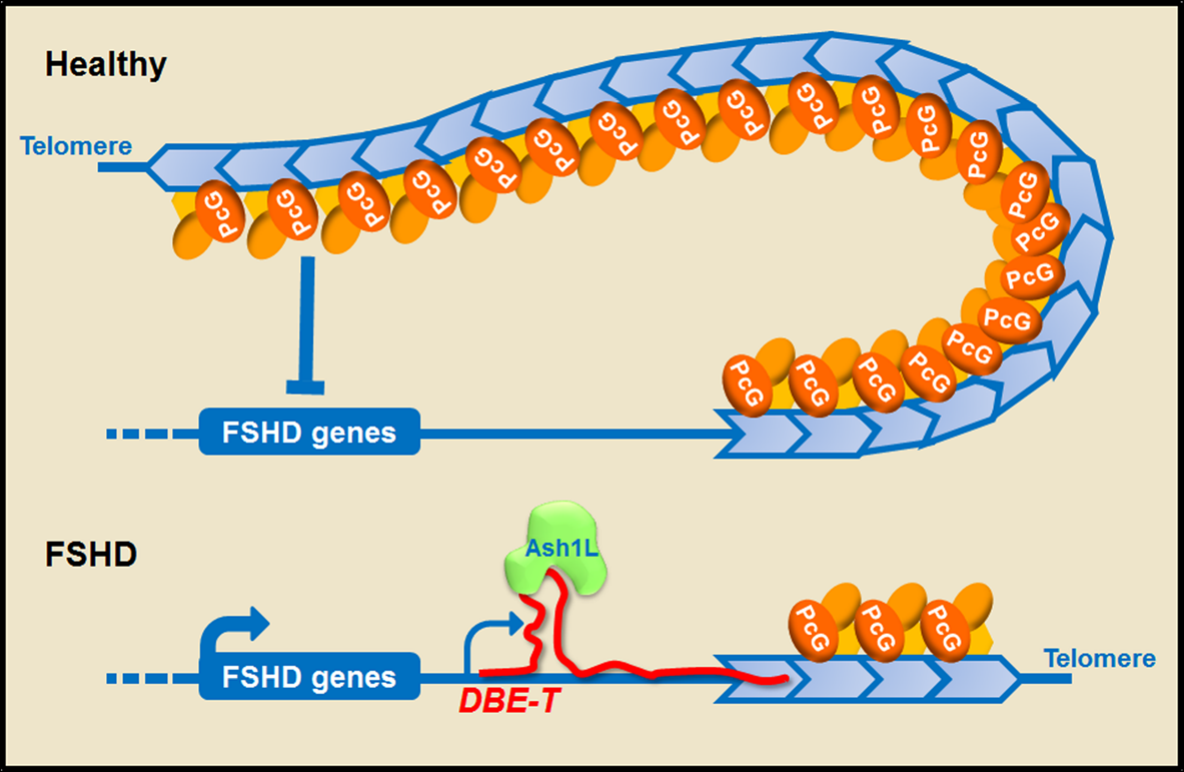
**Role of *DBE-T *long noncoding RNA (lncRNA) in facioscapulohumeral muscular dystrophy (FSHD).** In healthy individuals, the D4Z4 array displays from 11 to more than 100 units and is extensively bound by Polycomb group proteins (PcG), leading to the repression of the locus. In FSHD patients, the reduction of D4Z4 copy number to below 11 units causes decreased PcG binding and hence reduced silencing. This allows the transcription of the lncRNA *DBE-T* that remains associated to the FSHD locus and recruits the TrxG protein ASHL1 leading to activation of FSHD candidate genes.

Facioscapulohumeral muscular dystrophy (FSHD) is the third most common muscular dystrophy (1:14,000). FSHD is transmitted in an autosomal dominant manner and affects both sexes but presents a gender bias, as males are usually more severely affected [124]. FSHD displays a more restricted pattern of muscle weakness compared to DMD, mainly confined to the facial mimic and shoulder girdle muscles but extending to abdominal and leg muscles in the most severe cases [125,126]. The genetic lesion involved in FSHD is unusual as it does not target a protein-coding gene, but rather affects the copy number of the 3.3 kb macrosatellite D4Z4 mapping at the subtelomeric region of chromosome 4 (4q35) [127]. In the general population, D4Z4 copy number is highly polymorphic, displaying 11 to more than 100 units [128,129]. On the contrary, FSHD patients carry deletions reducing D4Z4 copy number between one and ten units [129,130]. D4Z4 deletion is associated with a profound change in the epigenetic status of the 4q35 region [131]. A recently identified lncRNA plays a key role in this transition [37]. In healthy subjects, the FSHD locus is under a repressive chromatin status, with high levels of DNA methylation, histone de-acetylation and enrichment for repressive histone marks such as H2Aub1, H3K9me3 and H3K27me3. Indeed, D4Z4 has been recently identified as a novel Polycomb (PcG) target region, suggesting that the presence of a high number of D4Z4 units leads to the extensive recruitment of PcG and the consequent repression of 4q35 genes in healthy subjects (Figure 4). In FSHD patients, instead, the reduction in number of D4Z4 units under a critical threshold leads to a reduced PcG binding with decreased levels of H3K27me3, particularly in the region immediately proximal to the D4Z4 repeat array. As a result, this region becomes more prone to transcription and gives rise to the activatory lncRNA *DBE-T. DBE-T* is mainly produced in FSHD patients and mediates the aberrant activation of the FSHD locus [37]. *DBE-T* is a nuclear transcript that acts *in cis* as it remains associated with the chromatin of the FSHD locus (Figure 4) [37]. *DBE-T* directly binds to the Trithorax (TrxG) protein ASHL1 recruiting it to the FSHD locus where it mediates the accumulation of H3K36me2 [37], a histone modification that counteracts PcG repressive activity [132-134]. Consequently, this leads to altered higher order chromatin organization and derepression of FSHD candidate genes localized nearby the D4Z4 array and unleashing FSHD pathogenesis [37].

## Conclusion

It is increasingly evident that the functional repertoire of metazoan genomes extends far beyond protein-coding genes. A growing body of genetic and biochemical work indicates that long noncoding RNAs are important members of the complex muscle regulatory network, being engaged in diverse activities crucial for myogenesis. However, there is still a substantial gap between the expanding list of muscle lncRNAs and the precise molecular tasks they fulfill in the control of muscle differentiation. Moreover, the functional characterization of lncRNAs in muscular dystrophy is still in its infancy. Nevertheless, there is little doubt that results from such studies will significantly contribute to the formulation of specific and complementary diagnostic and therapeutic strategies for muscle wasting diseases.

## Abbreviations

ceRNA: competing endogenous RNA; CERNA: *core enhancer RNA*; ChIP-seq: chromatin immunoprecipitation sequencing; DBE-T: D4Z4 binding element transcript; DMD: Duchenne muscular dystrophy; DMD: dystrophin gene; DRRRNA: *distal regulatory region RNA*; dsRNA: double-stranded RNA; eRNA: *enhancer RNA*; FSHD: facioscapulohumeral muscular dystrophy; Gtl2/Meg3: gene trap locus 2/maternally expressed gene 3; H3K27ac: acetylated histone H3 lysine 27; H3K4me1: monomethyl histone H3 lysine 4; IMP: Igf2 *mRNA binding-protein*; iPSC: induced pluripotent stem cell; IRES: internal ribosomal entry site; linc-MD1: long intergenic noncoding RNA-muscle differentiation 1; lincRNA: *long intergenic noncoding RNA*; lncRNA: long noncoding RNA; Malat1: *metastasis associated lung adenocarcinoma transcript 1*; MAML1: mastermind-like protein 1; MEF2: myocyte-specific enhancer factor-2; miRNA: microRNA; MRF4: myogenic regulatory factors; MyoD: myogenic differentiation; MyoG: myogenin; ncRNA: non protein-coding RNA; Nctc1: *noncoding transcript 1*; Neat1: *nuclear enriched abundant transcript 1*; NMD: nonsense-mediated mRNA decay; PcG: Polycomb group; piRNA: piwi-interacting RNA; PRC1 and PRC2: Polycomb Repressive Complex 1 and 2; RNAPII: RNA polymerase II; rRNA: ribosomal RNA; SINE: *short interspersed element*; siRNA: small interference RNA; SMD: Staufen-mediated mRNA decay; snoRNA: small nucleolar RNA; SNP: single nucleotide; snRNA: small nuclear RNA; SRA: *steroid receptor RNA activator*; SRAP: SRA protein; STAU1 and 2: Staufen 1 and 2; tRNA: transfer RNA; TrxG: Trithorax group; UTR: untranslated region; Yam: *YY1-associated muscle*; YY1: Yin Yang 1.

## Competing interests

The authors declare that they have no competing interests.

## Authors’ contributions

MVN: manuscript writing, final approval of the manuscript. MJ: manuscript writing, final approval of the manuscript. DG: manuscript writing, final approval of the manuscript. All authors read and approved the final manuscript.
